# Changes in the Professional Identity and Self-Efficacy of Nursing Students Engaged in the Objective Structured Clinical Examination Using Simulation Learning

**DOI:** 10.7759/cureus.68568

**Published:** 2024-09-03

**Authors:** Keisuke Nojima, Miki Morimoto

**Affiliations:** 1 Faculty of Nursing, Kyoto Tachibana University, Kyoto, JPN; 2 Nursing Department, Nishi Nara Central Hospital, Kyoto, JPN

**Keywords:** simulation-based education, nursing students, self-efficacy, professional identity, osce

## Abstract

Background: The Objective Structured Clinical Examination (OSCE) is widely adopted in nursing education to enhance clinical skills and professionalism. With OSCE, the learning process is important, and students who underwent OSCE felt more confident and better prepared for their next clinical training.

Objectives: This study aims to clarify how the self-efficacy and professional identity of nursing students change after learning through simulation education and OSCE. Clarification of these issues will allow an OSCE design utilizing simulation-based education as will be discussed.

Methods: This study used a pre-post study design, and the participants were 74 nursing university students at one university in Japan who agreed to participate in the study. The total scores and subscale scores for professional identity and self-efficacy were compared before and after the OSCE using the Wilcoxon signed-rank test. Spearman's rank correlation coefficient was calculated to examine the relationship between professional identity and self-efficacy.

Results: There were significant increases in self-efficacy scores (p<0.05) after OSCE, but there were no significant changes in the total scores of professional identities before and after the OSCE. Professional identity scores, such as choosing nursing again and desire to improve nursing skills, increased.

Conclusions: Simulation-based OSCE effectively enhances nursing students' self-efficacy and certain aspects of professional identity, indicating its potential for nursing education.

## Introduction

The Objective Structured Clinical Examination (OSCE) is an objective, structured, and organized evaluation framework designed to assess the comprehensive application of clinical skills and professionalism of clinical students through simulated clinical scenarios [1]. In Japan, nursing schools increasingly employ OSCE to enhance nursing practice skills.

The OSCE encompasses both formative and evaluative aspects, making it efficient as it can objectively assess the abilities of students, which are difficult to evaluate by other means [2]. Further, it has been concluded that OSCE is positively accepted by both students and faculty as a learning tool that provides feedback and helps students acquire necessary competencies [3]. In terms of validity, the OSCE method has been shown to ensure fairness and objectivity in evaluating communication skills throughout the examination period in large groups of nursing students for certification [4].

Despite being an objective assessment test, various innovations are incorporated into the OSCE learning process, showing multiple effects. For example, incorporating a flipped learning approach in an OSCE evaluating intravenous injection techniques fosters both nursing skills and critical thinking abilities [5]. Incorporating OSCE after lectures and core nursing skills practice improves the knowledge retention of nursing students, suggesting that OSCE enhances the clinical abilities of students [6].

Professional identity (PI) is defined as "an aspect of personal identity formation, which is a subjective feeling that is perceived in the interactions with the norms and value systems possessed by the occupational group." PI is a crucial element for students aspiring to be healthcare professionals like nurses. For nursing students, promoting PI is shown to effectively foster professional attitudes [7,8]. Enhancing PI of nurses is considered an effective method to reduce turnover rates [9], and PI has also been found to be a strong predictor of job retention in nursing [10]. This indicates that PI significantly impacts the ability of nurses to continue working without leaving the profession. Therefore, it is considered necessary to support the strengthening of PI from the student stage.

Reports on incorporating simulation education in medical schools suggest that simulation exercises can improve professional identity formation (PIF) and adding them longitudinally to the curriculum can further enhance PIF in medical students [11]. Self-efficacy (SE) is very important for learners to continue their studies. This is also true for nursing students, and various learning methods have been examined and discussed to enhance self-efficacy [12,13]. Further, reports indicate that higher levels of self-efficacy and social support can reduce academic burnout in nursing students [14], highlighting the importance of supporting self-efficacy in nursing students. However, a systematic review conducted to conceptualize the transition of nursing education and nursing students into nursing practice reported that various educational and learning interventions were used to enhance self-efficacy, but no definitive conclusions were drawn regarding the most effective educational intervention [15].

Recently, simulation techniques have been incorporated into nursing education. Simulation education is said to effectively develop nursing practice skills [16]. Additionally, engaging in simulation education involves aspects that enhance various learning attitudes and initiatives [17]. With OSCE, the learning process is important, and there are reports that students who underwent OSCE felt more confident and better prepared for their next clinical training [18]. Therefore, this study aims to clarify how the self-efficacy and PI of nursing students change after learning through simulation education and OSCE.

## Materials and methods

Participants

This study was conducted as a longitudinal survey study. In this study, the offer of participation in the study was emailed to 80 nursing students planning to take the OSCE at one university in Japan, and students who indicated their willingness to participate were given an explanation of the study and consent to participate. As a result, 74 nursing students were participants who had consented to take part in the study. Nursing universities in Japan provide four-year programs after which graduates take the national nursing examination. The sample size here was all seniors taking the OSCE during the study period. In examining the sample size, assuming a statistical power (power) of 80% and a medium effect size (Cohen's d=0.3), the required sample size was calculated to be approximately 175 participants. However, the sample size for this study was 74 participants, indicating that it was insufficient for the desired power. Therefore, type II errors should be noted in the interpretation of the results. Inclusion criteria included being in the final year of the nursing program and having completed all required clinical practicums. We determined responses without missing values to be valid and included them in the analysis. Exclusion criteria were students who had not completed all clinical practicums or who did not consent to participate in the study.

OSCE outline

The OSCE was conducted as an integrated practice course held just before graduation after completing all clinical practicums. The course consisted of 15 sessions of 90 minutes each. The test included "blood collection from hospitalized patients" and "initial response to a patient in shock." Before the exams for each item, students engaged in group learning of the knowledge and task simulations using simulators.

Data collection

Two anonymous questionnaire surveys were conducted with the study participants before and after the OSCE. The questionnaires included demographic attributes, a PI scale, and a self-efficacy scale.

PI scale

PI was assessed using 12 items created by Hatano and Onodera [19], answered on a five-point Likert scale from "strongly disagree" to "strongly agree." The total score for the 12 items used to assess PI ranged from 12 to 60, with higher scores indicating a stronger PI. No specific cut-off points have been set for the classification of scores. The items used in the PI scale are listed in Table 1.

**Table 1:** Professional identity scale

Item number	Item description
1	I hope to continue my nursing job for a long time in the future.
2	I think the job as a nurse is suitable for me.
3	If I had to choose a job again, I would choose nursing again.
4	If a high school student asked me if I wanted to be a nurse, I would recommend it.
5	I take pride in my nursing job.
6	I want to learn more about nursing.
7	I am satisfied with my nursing career choice.
8	I am confident in doing my job as a nurse.
9	I want to improve my nursing skills.
10	If my child wants to be a nurse, I would recommend it.
11	I think a job in nursing would be a great use of my talents.
12	I find my life's purpose in nursing.

Self-efficacy scale

The self-efficacy scale included nine items created by Mori [20], with responses on a seven-point Likert scale from "not at all true" to "very true." The total score for the nine items used in the self-efficacy scale ranges from 9 to 63, with higher scores indicating higher self-efficacy. No specific cut-off points have been established for the classification of scores. The items used in the self-efficacy scale are listed in Table 2.

**Table 2:** Self-efficacy scale

Item number	Item description
1	I think I will receive a good grade in this class.
2	I expect to do very well in this class.
3	I am sure I can do an excellent job in the problems and tasks assigned for this class.
4	I am certain I can understand the ideas taught in this course.
5	I know that I will be able to learn the material for this class.
6	Compared with other students in this class, I think I know a great deal about the subject.
7	Compared with other students in this class, I expect to do well.
8	Compared with others in this class, I think I'm a good student.
9	My study skills are excellent compared with others in this class.

Data analysis

Cronbach's alpha coefficient was calculated to examine the internal consistency of the scales used. PI and self-efficacy scores are not normally distributed, despite being continuous data. Therefore, the total scores and subscale scores for PI and self-efficacy were compared before and after the OSCE using the Wilcoxon signed-rank test. Spearman's rank correlation coefficient was calculated to examine the relationship between PI and self-efficacy. IBM SPSS Statistics for Windows, Version 27.0 (Released 2020; IBM Corp., Armonk, New York, United States) was used for the analysis, with a significance level of less than 5%.

Ethical considerations

Participation was not rewarded in any way and did not come with academic compensation. All participants received a written, verbal explanation of the study and were asked to respond freely and voluntarily. Throughout the study, data confidentiality and participant anonymity were ensured. This study was approved and conducted under the auspices of the Ethics Committee of Kyoto Tachibana University (approval number: 23-1).

## Results

Demographics

Of the 74 participants, valid responses from 66 participants (89.1%) without missing data were analyzed, as there were issues with the second round of responses. The breakdown by gender was 60 females (90.9%) and six males (9.1%).

Internal consistency verification

The reliability coefficients for the 12 items of the PI scale were high, with α=0.895 before OSCE and α=0.922 after OSCE. Similarly, the nine items of the self-efficacy scale also showed high reliability, with α=0.906 before OSCE and α=0.936 after OSCE, indicating internal consistency of both scales.

Changes in PI and self-efficacy before and after OSCE

There were no significant changes in the total scores of PI before and after the OSCE, but significant changes were observed in specific factors. Items such as "If I had to choose a job again, I would choose nursing again" (Z=-2.29, p=0.022) and "I want to improve my nursing skills" (Z=-4.58, p<0.001) showed significant increases (Table 3). Further, there were statistically significant differences in self-efficacy scores before and after the OSCE (Z=-2.31, p=0.021), with significant increases in items such as "I expect to do very well in this class" (Z=-2.65, p=0.008), "I am sure I can do an excellent job in the problems and tasks assigned for this class" (Z=-2.13, p=0.033), and "I know that I will be able to learn the material for this class" (Z=-2.09, p=0.036) (Table 4).

**Table 3:** Details of professional identity scores before and after intervention Wilcoxon signed-rank test; *: p<0.05

		Before intervention (n=66)		After intervention (n=66)		Z	P
		Median	IQR	Median	IQR		
	Total score	40.00	(2-4)	42.00	(2-5)	-1.61	0.11
1	I hope to continue my nursing job for a long time in the future.	4.00	(3-4)	4.00	(3-5)	-0.74	0.46
2	I think the job as a nurse is suitable for me.	3.00	(3-4)	3.00	(3-4)	-0.81	0.41
3	If I had to choose a job again, I would choose nursing again.	3.00	(2-3)	3.00	(2-4)	-2.29	0.02*
4	If a high school student asked me if I wanted to be a nurse, I would recommend it.	4.00	(3-4)	4.00	(3-4)	-1.39	0.16
5	I take pride in my nursing job.	4.00	(3-4)	4.00	(3-5)	-0.16	0.87
6	I want to learn more about nursing.	4.00	(3-4)	4.00	(3-4)	-0.72	0.47
7	I am satisfied with my nursing career choice.	4.00	(3-4)	4.00	(3-5)	-0.47	0.63
8	I am confident in doing my job as a nurse.	2.00	(2-3)	2.00	(2-3)	-0.56	0.58
9	I want to improve my nursing skills.	4.00	(3-4)	5.00	(4-5)	-4.58	0.00*
10	If my child wants to be a nurse, I would recommend it.	3.00	(3-4)	3.00	(3-4)	-0.17	0.86
11	I think a job in nursing would be a great use of my talents.	3.00	(3-4)	3.00	(2-4)	-0.17	0.86
12	I find my life's purpose in nursing.	3.00	(2-4)	3.00	(3-4)	-1.01	0.31

**Table 4:** Correlations between professional identity scores and self-efficacy scores before intervention Wilcoxon signed-rank test; *: p<0.05

		Before intervention (n=66)		After intervention (n=66)		Z	P
		Median	IQR	Median	IQR		
	Total score	30.00	(2-5)	34.00	(2-5)	-2.31	0.02*
1	I think I will receive a good grade in this class.	3.00	(2-4)	3.50	(2-4)	-0.37	0.71
2	I expect to do very well in this class.	3.00	(2-4)	4.00	(2-4)	-2.65	0.001*
3	I am sure I can do an excellent job in the problems and tasks assigned for this class.	3.00	(3-4)	4.00	(3-4)	-2.13	0.03*
4	I am certain I can understand the ideas taught in this course.	4.00	(3-5)	4.00	(3-5)	-1.70	0.09
5	I know that I will be able to learn the material for this class.	4.00	(3-5)	4.00	(3-5)	-2.09	0.03*
6	Compared with other students in this class, I think I know a great deal about the subject.	3.00	(2-4)	4.00	(4-5)	-2.85	0.001*
7	Compared with other students in this class, I expect to do well.	3.00	(2-4)	4.00	(3-4)	-1.60	0.10
8	Compared with others in this class, I think I'm a good student.	3.00	(2-4)	2.00	(3-4)	-1.36	0.17
9	My study skills are excellent compared with others in this class.	3.00	(2-4)	3.50	(2-4)	-1.52	0.12

Relationship between PI and self-efficacy

A significant positive correlation was observed between the total scores for PI and self-efficacy before OSCE (r=0.367, p=0.000), indicating a moderate correlation. Significant correlations were found in eight items of the subscales for PI and self-efficacy before OSCE, including "I think the job as a nurse is suitable for me" and "If I had to choose a job again, I would choose nursing again" (Table 5). Additionally, a significant positive correlation was observed between the total scores for PI and self-efficacy after OSCE (r=0.592, p=0.000). Significant correlations were found in all items of the subscales for PI and self-efficacy after OSCE (Table 6).

**Table 5:** Correlations between professional identity scores and self-efficacy scores before intervention *:p<0.01 (n=66)

		Self-efficacy scores
Professional identity score subscale		
1	I hope to continue my nursing job for a long time in the future.	0.180
2	I think the job as a nurse is suitable for me.	0.338*
3	If I had to choose a job again, I would choose nursing again.	0.291*
4	If a high school student asked me if I wanted to be a nurse, I would recommend it.	0.392*
5	I take pride in my nursing job.	0.123
6	I want to learn more about nursing.	0.198
7	I am satisfied with my nursing career choice.	0.370*
8	I am confident in doing my job as a nurse.	0.510*
9	I want to improve my nursing skills.	0.249*
10	If my child wants to be a nurse, I would recommend it.	0.246*
11	I think a job in nursing would be a great use of my talents.	0.386*
12	I find my life's purpose in nursing.	-0.035
Total score		0.367*

**Table 6:** Correlations between professional identity scores and self-efficacy scores after intervention *: p<0.01 (n=66)

		Self-efficacy scores
Professional identity score subscale		
1	I hope to continue my nursing job for a long time in the future.	0.329*
2	I think the job as a nurse is suitable for me.	0.567*
3	If I had to choose a job again, I would choose nursing again.	0.475*
4	If a high school student asked me if I wanted to be a nurse, I would recommend it.	0.445*
5	I take pride in my nursing job.	0.484*
6	I want to learn more about nursing.	0.367*
7	I am satisfied with my nursing career choice.	0.584*
8	I am confident in doing my job as a nurse.	0.492*
9	I want to improve my nursing skills.	0.371*
10	If my child wants to be a nurse, I would recommend it.	0.301*
11	I think a job in nursing would be a great use of my talents.	0.554*
12	I find my life's purpose in nursing.	0.496*
Total score		0.592*

Study applicability

The results of this study, although based on a sample size of 74 students from a single university, provide valuable insights into the impact of OSCE on PI and self-efficacy. The findings may be cautiously generalized to other nursing students in similar educational settings in Japan, particularly those nearing graduation after completing clinical practicums. However, further studies with larger and more diverse samples across multiple institutions are required to enhance the generalizability of the results.

## Discussion

Internal consistency of the PI scale and self-efficacy scale

The PI scale (α=0.895 before OSCE, α=0.922 after OSCE) and self-efficacy scale (α=0.906 before OSCE, α=0.936 after OSCE) in this study both showed values significantly exceeding the standard, indicating that these scales consistently provide reliable measurements, suggesting that the observed changes in PI and self-efficacy in this study can be interpreted as accurately reflecting these concepts. This enhances the reliability of the findings that OSCE significantly impacted nursing students' PI and self-efficacy.

Changes in PI and self-efficacy before and after OSCE

PI is a crucial factor in the education and career of nursing students. The formation of PI in nursing students is influenced by long-term clinical practice experience and the mentors involved [21,22]. Additionally, gaining self-esteem and confidence in practice positively impacts the formation of a PI [23]. In this study, OSCE incorporated simulation education, providing a learning experience that mimicked clinical nursing practices. This simulation experience would appear as offering opportunities for students to gain confidence through the reactions of the simulated patients and feedback from mentors, as well as to become aware of themselves as nurses. Although there were no significant changes in the overall scores of PI before and after OSCE, significant changes were observed in specific factors. The significant increase in items such as "If I had to choose a job again, I would choose nursing again" and "I want to improve my nursing skills" suggests that practical experiences with simulation education heightened student motivation and understanding of the nursing profession. Additionally, repeated simulation training before OSCE and receiving positive evaluations on their improvement probably made students more aware of their acquired skills, reinforcing their confidence in their career choice as nurses. Indeed, similar findings have been reported in previous studies, and there, simulation education significantly increased learners' confidence alongside knowledge improvement [24,25]. The lack of significant changes in other factors might be due to weaknesses in the simulation design or the limitations of the short-term simulated experiences.

Next, self-efficacy refers to the belief in one's own ability to handle difficult situations. The significant increase in self-efficacy scores after OSCE in this study suggests that the OSCE incorporating simulation education effectively enhanced student confidence and belief in their abilities. Self-efficacy profoundly influences individual behaviors, motivation, and persistence [26], and practical evaluation provides students with opportunities to acquire and confidently apply specific skills. The high scores on the self-efficacy subscales such as "I expect to do very well in this class," "I am sure I can do an excellent job in the problems and tasks assigned for this class," and "I know that I will be able to learn the material for this class" indicate that OSCE is recognized as an effective means to enhance the self-assessment abilities and practical problem-solving skills of students.

Additionally, Platt et al. [27] compared the effects of two educational designs based on immersive simulation on the knowledge and self-efficacy of nursing students, suggesting that repeated immersive simulation education, rather than a single instance of an experience, enhances self-efficacy. Similarly, in this study, students repeatedly practiced the knowledge and skills required for the exam before taking the OSCE, leading to attention to a sense of skill acquisition and increased self-efficacy.

From the above, although OSCE is an objective assessment test, the learning process leading up to the exam is crucial. Designing OSCE with repeated simulation training opportunities can enhance the PI and self-efficacy of nursing students. Future tasks include creating exercise designs that allow students to feel the specialty and attractiveness of nursing, as this study did not reach the level of improving PI.

Relationship between PI and self-efficacy

In this study, a weak positive correlation was observed between PI scores and self-efficacy scores before and after OSCE, indicating a slight relationship between the two. Students who evaluated their abilities highly before OSCE were more likely to find value in the nursing profession. Conversely, students with low self-efficacy may lack a clear purpose or direction in their studies. As previous studies have also stated that strategies to enhance PI are necessary from the early stages of nursing studies [28], these results suggest that early support for establishing PI is necessary.

The overall correlation increased from 0.367 before the OSCE to 0.592 after, emphasizing that OSCE effectively strengthened the relationship between nursing PI and self-efficacy. Specifically, the significant increases in the correlations of items such as "I think the job as a nurse is suitable for me" (0.567) and "I am satisfied with my nursing career choice" (0.584) suggest that the OSCE enhanced the student sense of career fit and satisfaction with their career choice, resulting in improving the self-efficacy. As previous studies have also stated that higher scores on self-efficacy were associated with the nursing student PI, students who studied in a good clinical learning environment had an indirect influence on student PI through self-efficacy [29]. In addition, students with high self-efficacy can improve their professionalism and self-confidence, thereby achieving high degrees of career maturity [30]; the correlation between PI scores and self-efficacy scores strengthened in all items, suggesting that the intervention positively impacted the relationship between these two components. These findings highlight the importance of using OSCE as a means of support that could be an efficient and beneficial strategy.

Limitations of the study

We believe that our sample is applicable mainly because the study targets general nursing training institutions in Japan. However, this study only investigated students at one university in Japan, and we do not expect the findings to be simply generalizable. Therefore, the findings need to be validated in the future by increasing the number of participants at a number of nursing training institutions.

## Conclusions

Although OSCE is an objective assessment test, designing an OSCE with repeated simulation training can potentially enhance the PI and self-efficacy of nursing students. However, this study did not achieve improvements in the PI, indicating the need for exercise designs that allow students to feel the specialty and attractiveness of nursing. Additionally, based on the results here, educators and clinical mentors should consider using OSCE as a strategy to enhance the nursing practice skills of nursing students.
